# Leveling the Playing Field: A New Proposed Method to Address Relative Age- and Maturity-Related Bias in Soccer

**DOI:** 10.3389/fspor.2021.635379

**Published:** 2021-03-04

**Authors:** Werner F. Helsen, Martine Thomis, Janet L. Starkes, Sander Vrijens, Gerrit Ooms, Calum MacMaster, Chris Towlson

**Affiliations:** ^1^KU Leuven, Department of Movement Sciences, Leuven, Belgium; ^2^Department of Kinesiology, McMaster University, Hamilton, ON, Canada; ^3^Department of Sport, Health and Exercise Science, University of Hull, Hull, United Kingdom

**Keywords:** relative age effect, maturity status, chronological birth date, developmental birth date, growth curve, allocation date, talent identification

## Abstract

Despite various solutions proposed to solve the relative age effect (RAE), it is still a major problem confounding talent identification and selection processes. In the first phase, we sampled 302 under 7–21 academy soccer players from two Belgian professional soccer clubs to explore the potential of a new approach to solve the inequalities resulting from relative age- and maturity-related bias. This approach allocates players into four discrete quartile groups based on the midway point of their chronological and estimated developmental (ED) birth dates (calculated using the growth curves for stature of Belgian youth). With the use of chi square analyses, a RAE was found (*p* < 0.01) for the overall sample (Q1 = 41.4% vs. Q4 = 14.9%) that completely disappeared after reallocation (Q1 = 26.5%; Q2 = 21.9%; Q3 = 27.5%; Q4 = 24.2%). According to the new allocation method, the stature difference was reduced, on average, by 11.6 cm (from 24.0 ± 9.9 to 12.4 ± 3.4 cm, *d* = 1.57). Body mass difference between the two methods was 1.9 kg (20.1 ± 11.3–18.2 ± 13.1 kg, respectively, *d* = 0.15). The new method created a maximum chronological age difference of 1.9 vs. 0.8 years for the current method. With the use of this method, 47% of the players would be reallocated. Twenty-three percent would be moved up one age category, and 21% would be moved down. In the second phase, we also examined 80 UK academy soccer players to explore if reallocating players reduces the within-playing group variation of somatic and physical fitness characteristics. The percentage coefficient of variation (%CV) was reduced (0.2–10.1%) in 15 out of 20 metrics across U11–U16 age categories, with the U13 age category demonstrating the largest reductions (0.9–10.1%) in CV. The U12 and U13 age categories and associated reallocation groupings showed *trivial* to *small* (ES = 0.0–0.5) between-method differences and *trivial* to *moderate* (ES = 0.0–1.1) differences within the U14–U16 age categories. A reduction in RAE may lead to fewer dropouts and thus a larger player pool, which benefits, in turn, talent identification, selection, and development.

## Introduction

The over-representation of soccer players born in the first 3 months (quartile) of the selection year is typically referred to as the relative age effect (RAE) (Cobley et al., 2009). This selection phenomenon has continued to confound talent identification since RAE was established (Barnsley et al., 1985). The RAE occurs within youth sports due to arbitrary annual age grouping [i.e., under (U)8, U9, and U10] with fixed cutoff dates in soccer that typically align with the calendar year (January 1 to December 31), except in the UK, where it is September 1 to August 30. Although these groupings are used to provide age-appropriate training and game formats, it does not account for the maturity-related differences within a given age category (Helsen et al., 2005) and can contribute to premature deselection and playing position allocation of soccer players (Towlson et al., 2017). Ultimately, it may confound the (de)selection processes that talent development centers employ, likely thwarting the size of the talent pool clubs that nations can select from.

The within age-group differences are a contributing factor for identifying players according to their relative (typically birth quartile), as opposed to chronological age. Categorizing players according to birth quartile has been shown to provide insight for practitioners when assessing anthropometrical and physical fitness-related characteristic differences within a chronologically categorized cohort of players (Cobley et al., 2009). This is of relevance and importance to talent development programs and national governing bodies, given that despite knowledge of the RAE spanning three decades (Helsen et al., 1998), a consistent over-representation of players born in the first and second quartiles of a selection year remains within development programs across the globe (Yagüe et al., 2020). The persistence of this within-year relative age selection phenomena is likely exacerbated by incentivized chronological age categorized match competitions (i.e., league tables and cups), spanning ages (between ~10.7 and ~15.2 years) associated with heightened periods of growth in stature [known as peak height velocity (PHV)] (Philippaerts et al., 2006; Towlson et al., 2018). In turn, this may result in large, temporary between-player maturity-related differences in physical and anthropometric characteristics, which may afford players born in quartiles 1 and 2 [who may also benefit from an early maturation (accelerated growth approximately 7.5 to 9.7 cm/year)] (Philippaerts et al., 2006; Towlson et al., 2018) a temporary physical and/or anthropometric advantage over their younger counterparts and likely further confound the (de)selection processes of practitioners (Philippaerts et al., 2006; Towlson et al., 2018). Obviously, these physical advantages often result in better performance and misrepresent the notion of “talent” at young ages. That said, some players born in the fourth quartile of the selection year may also be early developers and could potentially be compensated by advantages associated with advanced maturity that may result in perceived superior performance in “early competitive environments” and a subsequent selection bias in their favor (Helsen et al., 1998; Lovell et al., 2015; Yagüe et al., 2020).

In addition to within-year groups, the RAE can also transcend age groups establishing a between-year selection bias (Steingröver et al., 2017a). The between-year relative age selection phenomena have been shown to manifest in most national soccer teams and academy team selections, contributing typical over-selection (often four times more) of children born in the first quartile of the selection year in comparison with the last quartile (Barnsley et al., 1985; Barnsley and Thompson, 1988; Verhulst, 1992; Cobley et al., 2009; Nolan and Howell, 2010; Steingröver et al., 2017a). In current soccer systems, younger players are constantly trying to overcome selection bias throughout their development (particularly across adolescence), which is evidenced by the large dropout rates within popular team-sports such as soccer (Barnsley et al., 1985; Barnsley and Thompson, 1988; Verhulst, 1992; Helsen et al., 1998; Cobley et al., 2009; Nolan and Howell, 2010; Steingröver et al., 2017b). In addition, the between-year effect, which occurs when chronological age groups are aggregated (e.g., U10–U12–U14), (Schorer et al., 2013; Steingröver et al., 2017a), suggests that the older players are over-represented (e.g., in the U12 teams, there are more players who are 11 years old than players who are 10 years old).

Considering these findings, solutions to the RAE (Cobley et al., 2009) and maturity-associated selection biases (Cumming et al., 2017) have been suggested (for an overview of potential solutions, see Helsen and Starkes, 2020), which have included sport-specific cutoff dates (Musch and Hay, 1999; Musch and Grondin, 2001), nuanced talent identification strategies (Mann and van Ginneken, 2017), changing (Barnsley and Thompson, 1988; Helsen et al., 1998, 2012) and rotating the cutoff dates (Grondin et al., 1984; Barnsley and Thompson, 1988), accompanied by the Novem system, which implemented 9-month age categories (Boucher and Halliwell, 1991), and maturity status bio-banding (Cumming et al., 2018; Abbott et al., 2019; Romann et al., 2020; Towlson et al., 2020b). However, given the likely cross-age group disruption caused by some of the aforementioned interventions and the requirement for knowledge and experience in using complex maturity estimation algorithms (particularly when using bio-banding), it is perhaps not surprising that such interventions have failed in reducing the obvious persistence of the RAE and indeed maturity-selection bias worldwide, given the current rigid, chronologically aged ordered games and training programs within youth soccer (Yagüe et al., 2020). Therefore, the success of prospective interventions is seemingly highly dependent on complete league/national governing body support, which will permit flexibility for soccer academies to allocate players to a particular grouping on a semi-permanent [e.g., (half-) season long] with the option to systematically review the players' group status to an agreed schedule. Unfortunately, although different (but associated) (de)selection problems, the proposed “solutions” for reducing the RAE and/or the maturity selection bias have been ineffective thus far (although we acknowledge bio-banding research is its infancy). As a result, innovative, integrated research designs and strategies were proposed to eradicate such bias once and for all (Roberts et al., 2020). Therefore, the aim of this study was to propose and offer early examination of a new, relatively cheap, simple, and more practical way (i.e., absence of maturity estimation equations and/or player radiographs) ensuring that this new method of allocating youth players not solely using their chronological birth date, but also the estimated developmental (ED) birth date (based on their actual physical characteristics), is accessible to all levels of the soccer pyramid across the globe. Using two individual samples of academy soccer players, we specifically explored (1) if the midway point of the chronological and ED birth dates is an appropriate way to reallocate youth players (phase I) and (2) explore if the new method to reallocate players reduces the within-playing group variation of somatic and physical fitness characteristics within each group in comparison with traditional chronologically categorized categories using an independent sample of academy soccer players (phase II). This new cost-effective and simple method of reallocating players could potentially create a more stimulating climate for all players to develop throughout their career and decrease substantially the rate of dropout associated with the RAE.

## Methods

### Phase I: Examination of the Midway Point Between the Chronological and Estimated Developmental Birth Dates to Reallocate Youth Players

#### Participants

A convenience sample of 302 male academy soccer players (U7: *n* = 6; U8: *n* = 12; U9: *n* = 22; U10: *n* = 16; U11: *n* = 33; U12: *n* = 37; U13: *n* = 49; U14: *n* = 38; U15: *n* = 34; U16: *n* = 38; U18: *n* = 17), participating in two different Belgian professional soccer academies (indicated as Team X and Team Y) during the 2019–2020 domestic soccer season, were sampled. The oldest player was born on January 16, 2003, and the youngest player was born on May 16, 2013. The sample size was constrained by the finite number of players available to recruit from across the two academies involved. Informed and parental/guardian consent were acquired for each player prior to testing, and a detailed protocol (MP013675) was approved by the Research Ethics Committee UZ/KU Leuven, Belgium.

#### Procedure

In line with previous publications (Helsen et al., 2005; Steingröver et al., 2017a), players were grouped within each category according to birth day quartile (Q) (Q1: January 1 to March 31; Q2: April 1 to June 30; Q3: July 1 to September 30; and Q4: October 1 to December 31) and expressed as a percentage of the sample population. The mean (95% confidence interval [CI]) age, stature, and body mass difference (delta) per age category and per team (Table 1) were established according to chronological (Table 1A) and ED birth dates (Table 1A). The ED birth date was estimated by comparing the anthropometric characteristics of each player with the normative growth curves from a longitudinal study examining secular changes in biological maturation in Belgian boys of the same age categories (Roelants et al., 2009).

**Table 1A:** Number of players and mean (95% CI) anthropometric characteristics per year for team X.

**Year**	***n***	**Min stature (cm) (95% CI)**	**Max stature (cm) (95% CI)**	**Min body-mass (kg) (95% CI)**	**Max body-mass (kg) (95% CI)**
2003 (U18)	16	168.0 (165.5–170.6)	180.2 (177.1–183.4)	58.9 (56.6–61.1)	71.9 (69.2–74.5)
2004 (U16)	18	166.5 (164.0–169.1)	178.6 (174.5 to182.8)	53.0 (51.9–54.1)	68.0 (63.6–72.5)
2005 (U15)	19	161.2 (158.2–164.2)	173.3 (170.8–175.9)	48.9 (47.1–50.8)	62.4 (58.6–66.2)
2006 (U14)	16	152.4 (148.6–156.3)	168.8 (165.4–172.2)	39.9 (38.4–41.5)	53.5 (51.4–55.7)
2007 (U13)	22	147.8 (146.2 to149.4)	157.8 (154.9 to160.6)	35.9 (35.2–55.6)	45.3 (43.4–47.1)
2008 (U12)	22	145.4 (143.5–147.2)	153.6 (152.0–155.2)	34.7 (34.3–35.2)	39.9 (39.4–40.5)
2009 (U11)	25	137.9 (136.3,139.5)	146.0 (144.9 to147.1)	30.7 (30.0–31.3)	37.4 (37.0–37.8)
**Total**	**138**	**152.4 (149.8–155.1)**	**164.1 (160.9–167.1)**	**41.7 (39.3–44.01)**	**52.6 (49.4–55.8)**

*Key: Min, minimum; Max, maximum; CI, confidence interval; Kg, kilogram; cm, centimeter*.

#### Anthropometric Measures

Players' date of birth, the measurement date, and decimal age at the time of measurement were collected for each player. With the use of previously outlined procedures (Towlson et al., 2017), duplicate measurements of stature, seated height (0.1-cm precision, Seca^©^ Portable Stadiometer, Hamburg, Germany), and body mass (0.1 kg, Seca^©^, Hamburg, Germany) of each player were collected. These measurements were performed by two certified practitioners of the respective clubs as part of their normal sports science monitoring. If the measurements varied ≥0.4 cm or 0.4 kg, a third measure was taken, and the median value recorded. Estimated leg length was recorded as stature minus seated height. Tables 1A,B provide an overview of the mean (95% CI) anthropometric characteristics per year for Team X and Team Y, respectively.

**Table 1B:** Number of players and mean (95% CI) anthropometric characteristics per year for team Y.

**Year**	***n***	**Min stature (cm) (95% CI)**	**Max stature (cm) (95% CI)**	**Min body-mass (kg) (95% CI)**	**Max body-mass (kg) (95% CI)**
2003 (U18)	1	174.5 (/)	174.5 (/)	56.8 (/)	56.8 (/)
2004 (U16)	20	166.6 (163.1–170.2)	178.2 (176.7–179.6)	54.1 (50.7–57.6)	71.6 (67.8–75.5)
2005 (U15)	15	162.6 (157.9–167.2)	178.0 (173.3–182.6)	81.0 (77.2–84.7)	90.2 (87.5–93.0)
2006 (U14)	22	154.4 (153.0 to155.7)	164.12 (160.9–167.3)	42.5 (41.4–43.7)	50.6 (47.6–53.5)
2007 (U13)	27	144.2 (142.2–146.3)	160.8 (157.1–164.5)	34.3 (32.9–35.6)	48.7 (44.4–52.9)
2008 (U12)	15	146.9 (144.4–149.4)	154.1 (151.1–157.0)	34.1 (33.1–35.2)	40.3 (37.9–42.6)
2009 (U11)	8	128.0 (123.92–132.08)	139.5 (133.0–145.9)	26.0 (23.0–28.9)	32.9 (29.9–35.9)
2010 (U10)	16	133.00 (130.3–135.7)	141.6 (139.0–143.6)	27.5 (25.9–29.2)	33.3 (31.7–34.8)
2011 (U9)	22	126.1 (124.23, 127.95)	135.2 (133.3, 137.1)	24.4 (23.6, 25.1)	29.0 (27.9, 30.0)
2012 (U8)	12	123.17 (121.4, 125.0)	126.3 (123.9, 128.8)	22.2 (21.6, 22.8)	25.6(24.2, 27.1)
2013 (U7)	6	112.00 (109.2, 114.8)	122.7 (119.2, 126.2)	19.0 (18.7, 19.4)	22.2(20.6, 23.8)
**Total**	**164**	**133.4 (131.4–135.4)**	**164.2 (162.0–166.4)**	**28.3 (27.3–29.4)**	**57.3 (53.6–61.0)**

*Min, minimum; Max, maximum; CI, confidence interval; Kg, kilogram; cm, centimeter*.

#### New Player Reallocation Method

**Figure 1:**
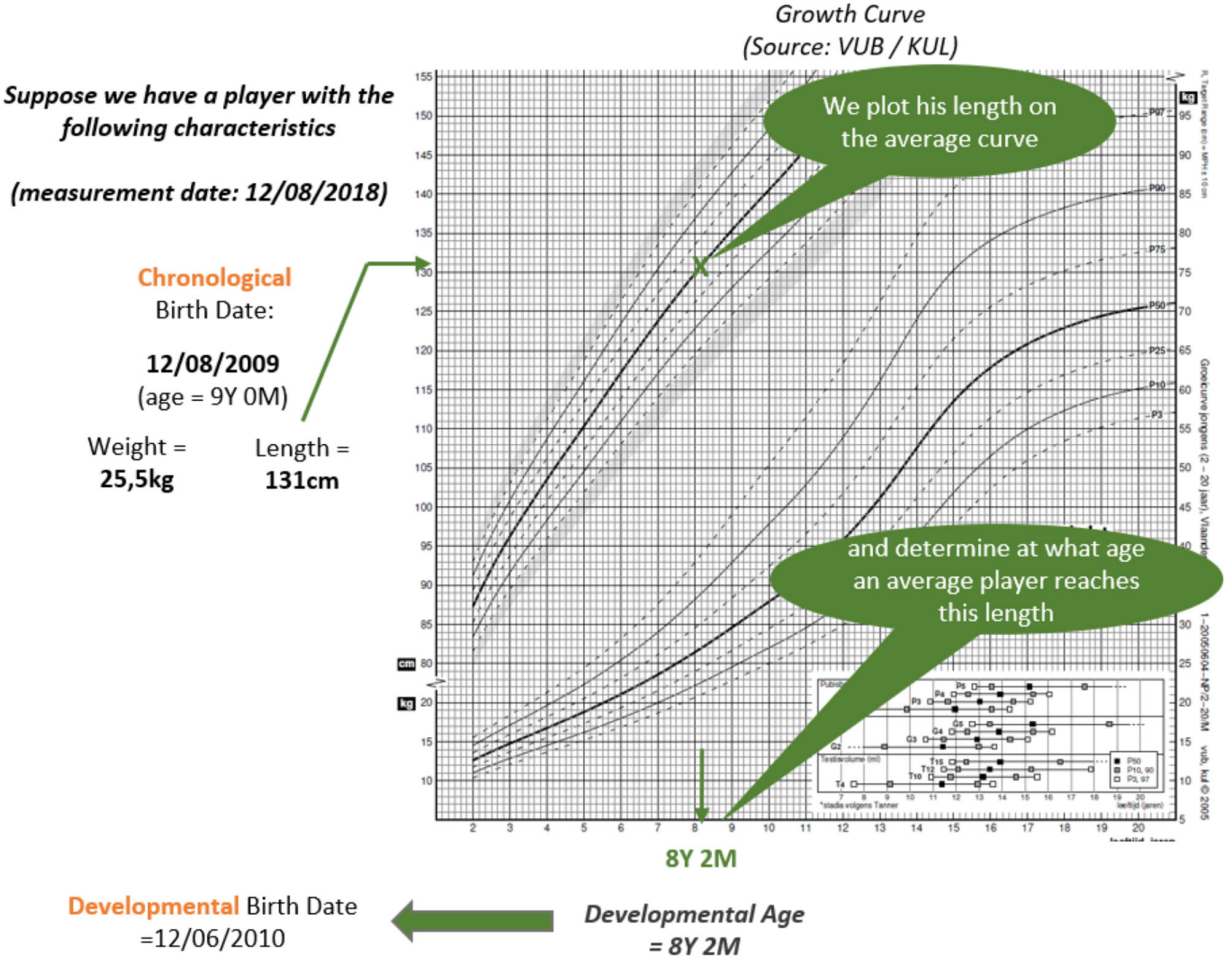
Using the 50th percentile of the normal weight growth curve of the Belgian population (Roelants et al., 2009), the estimated developmental birth date was determined by plotting the player's stature on the corresponding 50th percentile curve to establish the player's estimated developmental age for stature.

With the use of the 50th percentile of the normal weight growth curve of the Belgian population (Roelants et al., 2009), the ED birth date was determined. This was established by plotting each player's stature on the corresponding 50th percentile curve to determine the player's ED age for stature (e.g., if a male player was 131-cm tall, his ED birth date is 8 years 2 months because 50% of the children are 131-cm tall considering a 1-month scale) (see Figure 1). Given that stature and body mass are highly correlated in this sample (*r* = 0.876; *p* < 0.01), only stature was used within the new allocation method. We acknowledge that during ages associated with post PHV, muscle mass develops at a faster tempo than stature (Towlson et al., 2018), likely due to enhanced levels of muscle growth hormones (Malina et al., 2004), accompanied by a greater proportion of training dedicated to developing strength and power (Ford et al., 2011). However, such enhanced rates in body mass growth (i.e., body mass > stature) likely coincide with a limited number of age categories (U16–U18) toward the upper end of our sampled population. Therefore, only stature was considered as the main reference point for our new allocation method in order to maintain simplicity, as this was considered a key objective in an attempt to reduce cross-age group disruption to games and training programs. Because the ED birth date could theoretically lead to age differences of up to 5 years with the child's chronological age (as will be shown below), the *median birth date* was calculated between the chronological and ED birth dates, which represented the new allocation date that was used to (re)allocate each player into a younger or older category or maintain the same age category. For instance, a U10 player with a calculated allocation date of 05/06/2011 was allocated to the U9 category. However, if the allocation date was 05/06/2009, then the player would be allocated to the U11 category. A customized spreadsheet was used to calculate the ED birth date. Following this, the number of players who either were reallocated (to either a higher or a lower team) or remained in the same age category was established, and a comparison was made between the aforementioned differences in the current selection method and the new allocation method with respect to stature, body mass, and age.

**Figure 2:**
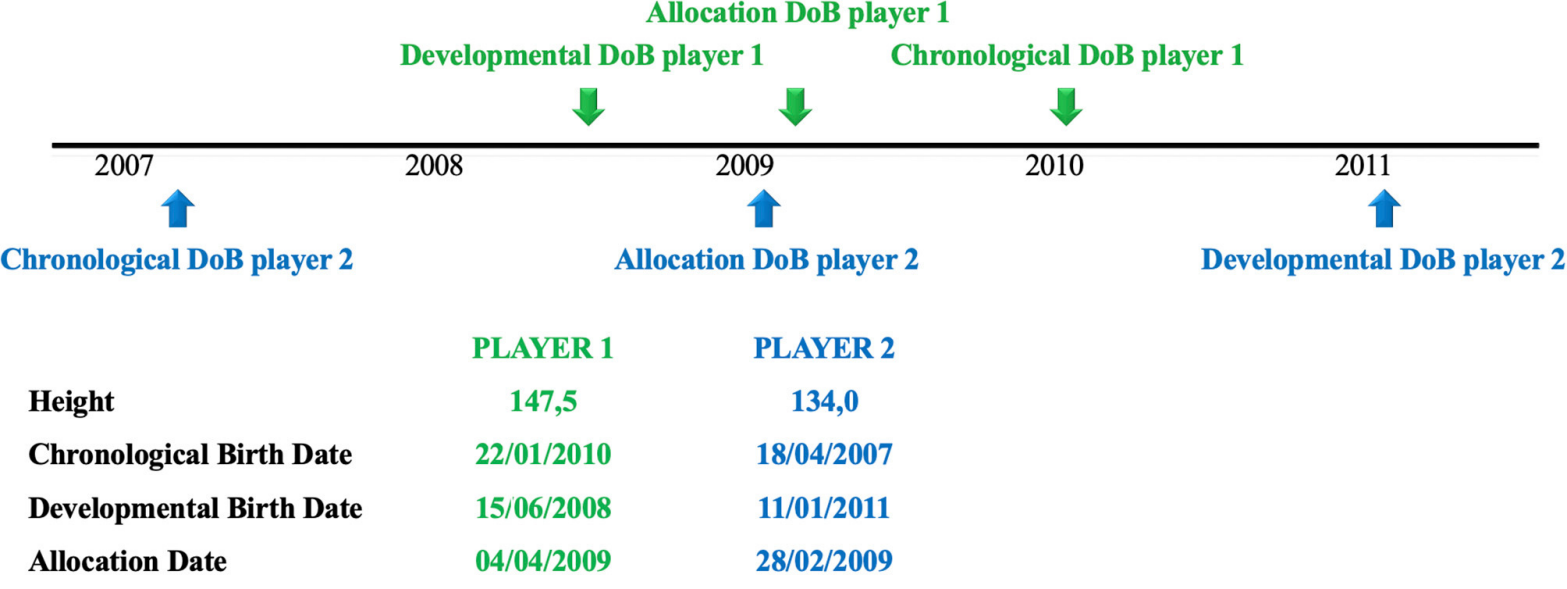
Outcome of the new method that prescribes an appropriate developmental allocation date. Player 1 is currently playing in the U10 category and player 2 in the U12 category. After reallocation they are both allocated in the U11 category.

If the allocation date was based solely on ED birth date, then the age difference would become much larger. On average, there would be a 3.3-year difference between players in the same team, and the stature difference would be 6 cm on average. This could decrease the advantage for the younger player being the tallest because the oldest player could be more than 5 years older and just 5.5 cm shorter in the most extreme case as illustrated in Figure 2. The proposed new method for allocating players is expected to create a more “level playing field” by reducing the within-group variation of somatic and physical fitness characteristics, as its proposed that the new method will afford smaller players the opportunity to develop their talents in a fair way.

### Phase II: Assessment of the Within-Group Variation of Somatic and Physical Fitness Characteristics Using Chronological and Estimated Developmental Birth Dates

#### Anthropometric and Maturation Status Measures

As stated in phase I, a convenience sample of 80 academy soccer players (U12: *n* = 18; U13: *n* = 14; U14: *n* = 15; U15: *n* = 17; U16: *n* = 16), participating in one UK professional soccer academy during the 2019–2020 domestic soccer, were considered. Player mean (95% CI) age, stature, and body mass difference (delta) per chronological age category (**Table 6**) and reallocation group (accompanied by ED birth data) were established using methods outlined in phase I. The same anthropometric variables from each player were recorded by one certified practitioner as stated in phase I (Towlson et al., 2017). Self-reported parental stature of both biological parents was also collected using previously outlined procedures (Cumming et al., 2018). To estimate maturation status, both biological parents self-reported their stature, which was adjusted for over-estimations using validated equation (Malina et al., 2007) based on measured and self-reported stature of US adults (Epstein et al., 1995), which provided an estimated percentage of final adult stature attainment (%EASA), commonly used in academy soccer research (Abbott et al., 2019; Towlson et al., 2020a,b).

#### Physical Fitness Measures

Players performed a battery of field tests to reflect the level of perceived importance placed on power, acceleration speed, and agility by talent practitioners when considering physical qualities of academy soccer players during the selection process (Deprez et al., 2015; Towlson et al., 2019). Explosive lower limb power was assessed using a vertical counter-movement jump (CMJ) (Optojump, Microgate, Bolzano, Italy), according to previously outlined methods (Tanner and Gore, 2012). As per our previous studies (Towlson et al., 2017), players performed two CMJs interspaced by 1 min of passive recovery; and if the difference in jump heights differed more than 2 cm, a third jump was recorded (maximum of eight jumps) with the mean of the highest three jumps be recorded. Players also performed a stationary start, running acceleration test over 20 m using previously established methods (Tanner and Gore, 2012), which was expressed as the time taken to complete each split (i.e., 0–5, 0–10, and 0–20 m) using digital timing gates (Brower Timing System, Salt Lake City, Utah, USA). Players performed three repetitions, and the best time was recorded interspace by 3 min of passive recovery. Lastly, players' agility performance was assessed using the 5–0–5 agility test (Draper, 1985). From a stationary starting position, players were required to maximally accelerate for 15 m and then turn 180° and accelerate back to the start–finish line. The time taken to complete the final and first 5 m of each leg (10 m in total) was recorded using digital timing gates (Brower Timing System, Salt Lake City, Utah, USA). Players performed two repetitions, turning off each leg, and the best time was recorded.

#### Statistical Analyses

For phase I, a statistical analysis of the RAE was completed for the whole dataset as well as per age category. The statistical analyses included chi square, Kolmogorov–Smirnov, and a regression analysis. Because of the relatively small sample sizes especially in the younger categories, the age categories were grouped by aggregating two categories (i.e., U7 and U8). Mean and 95% CI were calculated for player stature and body mass. Effect sizes were calculated using Cohen's *d*, as appropriate. Cohen's *d* values for *small, medium*, and *large* effects are 0.20, 0.50, and 0.80, respectively (Cohen, 1988). Significance level for all tests was set at *p* < 0.05.

For phase II, the within-group variation for chronological and reallocation methods players, the percentage coefficient of variation (%CV) was calculated as the standard deviation of the between-trial difference, divided by the mean between-trial difference. Data were presented as mean (95% CI) for each grouping; and Cohen's *d* values for *small, medium*, and *large* effects are 0.20, 0.50, and 0.80, respectively (Cohen, 1988), established for chronological vs. reallocation groupings with the accompanying qualities: trivial (<0.20), small (>0.21–0.60), moderate (>0.61–1.20), large (>1.21–2.00), and very large (>2.01) (Hopkins et al., 2009).

## Results

### Results (Phase I)

#### Relative Age Effect

**Figure 3:**
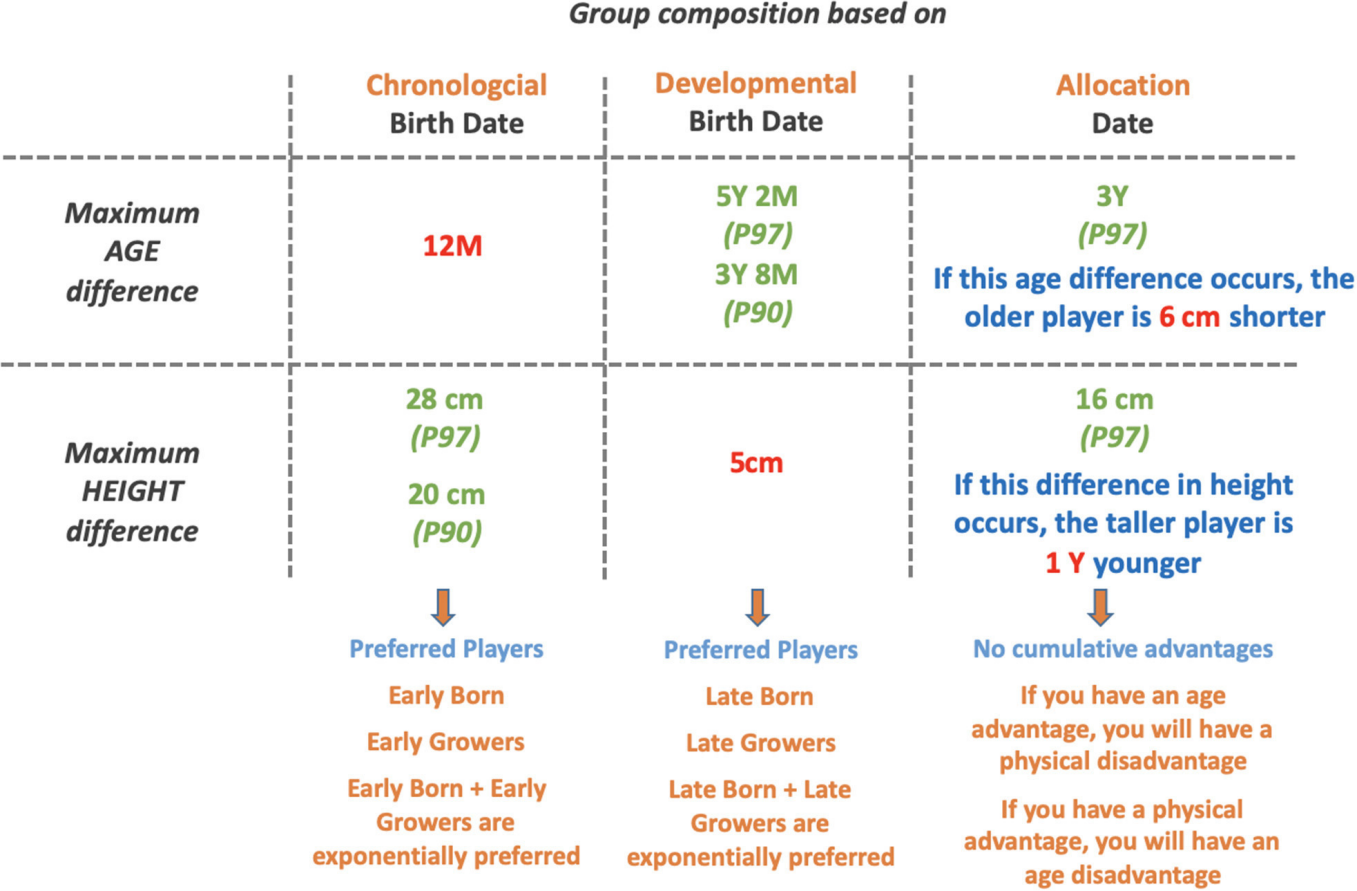
Differences in age and physical development of U10 players for group compositions based on chronological birth date, developmental birth date, and allocation date.

Figure 3 shows the birth date distribution per team and per quarter for all age categories before and after reallocation.

For the total dataset before reallocation (Figure 3A), there was a significant difference in distribution of players between the observed and expected birth date distributions (based on the Belgian population for the corresponding years). Most players were born in the first quarter (χ^2^ = 53.48; *p* < 0.01). This result was also supported by the result of a Kolmogorov–Smirnov analysis (*p* < 0.01) and a regression analysis (*r* = −0.84; *p* < 0.01). The aggregated age groupings (i.e., U7–U8 and U9–U10) demonstrated that a RAE was present compared with the population norm distributions (*p* < 0.01; Q1 _=_ 44.4 and 36.8%; Q4 = 5.6–10.5%) except for the U11–U12 group (χ^2^ = 5.06; *p* > 0.05; *r* = −0.55 *p* < 0.05; Q1 = 35.7%; Q4 = 18.6%). Table 2 shows the anthropometrical characteristics of each team according to age category accompanied by the mean (95% CI) and deltas. The difference between the tallest and smallest players in the younger categories was 18.0 (U7), 10.0 (U8), 22.0 (U9), 20.5 (U10), 29.0 (U11), and 23.6 cm (U12) and was enhanced within the older age categories to 38.7 (U13), 33.6 (U14), 42.6 (U15), 34.5 (U16), and 24.7 cm (U18). The mean difference between minimal and maximal age is 0.8 years; for stature, it is 24.0 cm; and the mean difference in body mass was 20.1 kg. Stature and body mass demonstrated a *strong* correlation (*r* = 0.876; *p* < 0.01).

**Table 2:** Overview of the anthropometrical and date of birth characteristics of 302 Belgian academy soccer players per age category for Team X and Y before reallocation.

**Year**	***n***	**(Min stature (cm) % CI)**	**Max stature (cm) (95% CI)**	**Min body-mass (kg) (95% CI)**	**Max body-mass (kg) (95% CI)**	**Min DoB**	**Max DoB**	**Delta age (years)**	**Delta stature (cm)**	**Delta body-mass (kg)**
**2003**	**17**	**168.73 (166.1, 171.4)**	**179.60 (176.6, 182.6)**	**58.63 (56.53, 60.74)**	**71.04 (68.46, 73.63)**	**16/01/2003**	**3/12/2003**	**0.9**	**24.7**	**26.5**
Team X	16	168.01 (165.5, 170.6)	180.24 (177.1, 183.4)	58.86 (56.63, 61.10)	71.85 (69.20, 74.50)	16/01/2003	3/12/2003	0.9	24.7	26.5
Team Y	1	174.5 (/)	174.5 (/)	56.8(/)	56.8(/)	11/08/2003	11/08/2003	0.0	0.0	0.0
**2004**	**38**	**166.43 (164.3, 168.6)**	**178.52 (176.5, 180.5)**	**53.52 (52.04, 54.99)**	**70.02 (67.10, 72.93)**	**7/01/2004**	**31/12/2004**	**1.0**	**34.5**	**38.1**
Team X	18	166.53 (164.0, 169.1)	178.63 (174.5,182.8)	53.03 (51.99, 54.08)	166.53 (164.02, 169.05)	7/01/2004	16/12/2004	0.9	34.2	35.3
Team Y	20	166.61 (163.1, 170.2)	178.15 (176.7, 179.6)	54.14 (50.69, 57.59)	71.61 (67.76, 75.46)	29/01/2004	31/12/2004	0.9	24.6	38.1
**2005**	**34**	**161.62 (159.1, 164.2)**	**175.56 (172.9, 178.2)**	**53.69 (51.23, 56.16)**	**84.26 (81.63, 86.89)**	**2/01/2005**	**21/12/2005**	**1.0**	**42.6**	**57.3**
Team X	19	161.19 (158.2, 164.2)	173.33 (170.8, 175.9)	48.96 (47.11, 50.81)	62.41 (58.65, 66.17)	14/01/2005	21/12/2005	0.9	28.0	33.5
Team Y	15	162.55 (157.9, 167.2)	177.95 (173.3, 182.6)	80.98 (77.22, 84.73)	90.21 (87.48, 92.95)	2/01/2005	7/08/2005	0.6	41.2	30.2
**2006**	**38**	**153.41 (151.6, 155.2)**	**166.25 (163.8, 168.7)**	**41.36 (40.49, 42.24)**	**51.88 (49.80, 53.97)**	**1/01/2006**	**11/12/2006**	**0.9**	**33.6**	**26.4**
Team X	16	152.41 (148.6, 156.3)	168.84 (165.4, 172.2)	39.96 (38.43, 41.49)	53.51 (51.38, 55.65)	4/01/2006	23/10/2006	0.8	33.6	25.9
Team Y	22	154.37 (153.0, 155.7)	164.12 (160.9, 167.3)	42.52 (41.37, 43.67)	50.56 (47.64, 53.49)	1/01/2006	11/12/2006	0.9	22.2	21.3
**2007**	**49**	**145.73 (144.2, 147.2)**	**159.52 (157.1, 162.0)**	**34.94 (34.24, 35.65)**	**47.18 (44.62, 49.74)**	**1/01/2007**	**27/11/2007**	**0.9**	**38.7**	**33.1**
Team X	22	147.76 (146.2,149.4)	157.79 (155.0,160.6)	35.85 (35.20, 55.65)	45.28 (43.44, 47.12)	2/01/2007	29/10/2007	0.8	24.6	19.4
Team Y	27	144.24 (142.2, 146.3)	160.77 (157.1, 164.5)	34.26 (32.89, 35.63)	48.65 (44.44, 52.86)	1/01/2007	27/11/2007	0.9	38.7	33.1
**2008**	**37**	**145.98 (144.4, 147.5)**	**153.79 (152.3, 155.3)**	**34.48 (33.90, 35.07)**	**40.11 (39.11, 41.10)**	**11/01/2008**	**25/12/2008**	**1.0**	**23.6**	**15.1**
Team X	22	145.35 (143.5, 147.2)	153.59 (152.0, 155.2)	34.75 (34.33, 35.18)	39.95 (39.38, 40.53)	13/01/2008	25/12/2008	1.0	19.6	10.8
Team Y	15	146.85 (144.4, 149.4)	154.06 (151.1, 157.0)	34.14 (33.05, 35.24)	40.26 (37.87, 42.64)	11/01/2008	28/09/2008	0.7	23.6	14.8
**2009**	**33**	**134.52 (132.3, 136.7)**	**145.52 (144.3, 146.8)**	**29.23 (28.44, 30.02)**	**36.71 (34.83, 38.58)**	**2/01/2009**	**30/12/2009**	**1.0**	**29.0**	**27.9**
Team X	25	137.91(136.3,139.5)	146.00 (144.9,147.1)	30.68 (30.03, 31.34)	37.42 (37.01, 37.84)	2/01/2009	25/11/2009	0.9	16.8	22.3
Team Y	8	128.00 (123.9, 132.1)	139.48 (133.0, 145.9)	25.95 (22.96, 28.94)	32.93 (29.93, 35.92)	19/01/2009	30/12/2009	0.9	29.0	16.2
**2010**	**16**	**133.00 (130.3, 135.7)**	**141.63 (139.7, 143.6)**	**27.53 (25.90, 29.15)**	**33.29 (31.74, 34.84)**	**12/01/2010**	**4/11/2010**	**0.8**	**20.5**	**12.5**
Team Y	16	133.00 (130.3, 135.7)	141.63 (139.7, 143.6)	27.53 (25.90, 29.15)	33.29 (31.74, 34.84)	12/01/2010	4/11/2010	0.8	20.5	12.5
**2011**	**22**	**126.09 (124.2, 128.0)**	**135.18 (133.3, 137.1)**	**24.36 (23.64, 25.08)**	**28.98 (27.94, 30.02)**	**3/01/2011**	**29/11/2011**	**0.9**	**22.0**	**9.7**
Team Y	22	126.09 (124.2, 128.0)	135.18 (133.3, 137.1)	24.36 (23.64, 25.08)	28.98 (27.94, 30.02)	3/01/2011	29/11/2011	0.9	22.0	9.7
**2012**	**12**	**123.17 (121.4, 125.0)**	**126.33 (123.9, 128.8)**	**22.17 (21.59, 22.75)**	**25.62 (24.15, 27.09)**	**16/01/2012**	**25/10/2012**	**0.8**	**10.0**	**7.6**
Team Y	12	123.17 (121.4, 125.0)	126.33 (123.9, 128.8)	22.17 (21.59, 22.75)	25.62 (24.15, 27.09)	16/01/2012	25/10/2012	0.8	10.0	7.6
**2013**	**6**	**112.00 (109.2, 114.8)**	**122.67 (119.2, 126.2)**	**19.03 (18.65, 19.42)**	**22.17 (20.58, 23.76)**	**9/01/2013**	**16/05/2013**	**0.3**	**18.0**	**4.7**
Team Y	6	112.00 (109.2, 114.8)	122.67 (119.2, 126.2)	19.03 (18.65, 19.42)	22.17 (20.58, 23.76)	9/01/2013	16/05/2013	0.3	18.0	4.7
**Total**	**302**	**139.10 (137.5, 140.7)**	**166.84 (165.5, 168.2)**	**31.59 (30.73, 32.44)**	**57.67 (55.70, 59.65)**	**16/01/2003**	**16/05/2013**	**0.8**	**24.0**	**20.1**

#### Effect of Reallocation

Following application of the new reallocation method (Figure 3B), differences disappeared for the number of players per birth date quarter as shown in Figure 3B. For both teams, each quarter was now composed of approximately 25% of the players (Q1 = 26.5%; Q2 = 21.9%; Q3 = 27.5%; Q4 = 24.2%).

In Table 3, the newly allocated teams are shown. Again, data are divided per team, and the deltas for age, stature, and body mass are given. The stature and body mass differences became greater with age within each group. The average deltas are now 1.9 years, 12.4 cm, and 18.2 kg.

**Table 3:** Physical characteristics of 302 Belgian academy soccer players, per age category and per team after reallocation.

**Year**	***n***	**Min stature (cm)(95% CI)**	**Max stature (cm) (95% CI)**	**Min body-mass (kg) (95% CI)**	**Max body-mass (kg) (95% CI)**	**Min DoB**	**Max DoB**	**Delta age (years)**	**Delta stature (cm)**	**Delta body-mass (kg)**
**2002**	**5**	**183.47 (182.0, 184.9)**	**188.23 (184.4, 192.1)**	**71.87 (70.1, 73.7)**	**79.27 (74.7, 83.8)**	**16/01/2003**	**7/01/2004**	**1.0**	**10.9**	**13.1**
Team X	5	183.47 (182.0, 184.9)	188.23 (184.4, 192.1)	71.87 (70.1, 73.7)	79.27 (74.74, 83.79)	16/01/2003	7/01/2004	1.0	10.9	13.1
**2003**	**21**	**172.94 (171.8, 174.1)**	**177.92 (176.3, 179.5)**	**63.45 (61.0, 66.0)**	**77.28 (71.3, 83.3)**	**31/01/2003**	**30/07/2005**	**2.5**	**20.9**	**43.8**
Team X	9	174.96 (173.6, 176.4)	180.38 (178.6, 182.2)	63.50 (59.4, 67.6)	69.68 (68.1, 71.2)	31/01/2003	26/06/2004	1.4	10.2	18.0
Team Y	12	177.13 (176.0, 178.2)	182.80 (178.9, 186.7)	63.68 (60.4, 67.0)	83.4 (75.2, 91.5)	11/08/2003	30/07/2005	2.0	19.0	41.7
**2004**	**34**	**165.56 (164.0, 167.1)**	**173.44 (172.2, 174.7)**	**55.47 (54.3,56.6)**	**68.78 (63.9,73.7)**	**6/03/2003**	**12/01/2006**	**2.9**	**17.1**	**43.6**
Team X	23	168.06 (166.5, 169.6)	175.02 (173.5, 176.5)	55.58 (54.4, 56.7)	64.40 (61.35, 67.45)	6/03/2003	12/01/2006	2.9	17.1	22.3
Team Y	11	171.87 (170.2, 173.5)	175.25 (174.6, 175.9)	55.33 (52.7, 58.0)	77.43 (67.0, 87.9)	4/02/2004	7/08/2005	1.5	8.8	43.6
**2005**	**33**	**157.71 (156.5, 159.0)**	**167.40 (166.4, 168.4)**	**50.68 (49.1,52.3)**	**69.56 (63.7,75.5)**	**4/02/2004**	**5/03/2007**	**3.1**	**14.2**	**42.3**
Team X	17	163.57 (162.1, 165.1)	169.72 (168.9, 170.6)	50.70 (49.4, 52.0)	59.22 (55.4, 63.0)	4/02/2004	23/10/2006	2.7	12.7	27.1
Team Y	16	162.74 (160.5, 165.0)	170.14 (168.9, 171.4)	51.36 (47.8, 55.0)	80.50 (74.8, 86.2)	15/05/2004	5/03/2007	2.8	14.2	42.3
**2006**	**37**	**151.41 (150.3, 152.5)**	**160.21 (158.5, 161.9)**	**43.42 (42.5,44.3)**	**56.78 (51.6,62.0)**	**8/01/2005**	**27/11/2007**	**2.9**	**17.2**	**44.6**
Team X	14	155.86 (153.7, 158.0)	163.14 (161.4, 164.9)	41.77 (40.3, 43.2)	48.80 (46.7, 50.90)	17/02/2005	14/10/2007	2.7	16.7	14.4
Team Y	23	155.76 (154.7, 156.8)	163.21 (161.2, 165.2)	44.64 (43.7, 45.6)	61.17 (54.1, 68.3)	8/01/2005	27/11/2007	2.9	15.8	41.5
**2007**	**41**	**149.55 (148.5, 150.6)**	**155.84 (154.6, 157.1)**	**36.57 (35.8, 37.4)**	**42.88 (41.5,44.2)**	**4/01/2006**	**22/09/2008**	**2.7**	**18.1**	**16.8**
Team X	24	149.13 (147.7, 150.6)	155.90 (154.4, 157.4)	36.48 (35.6, 37.3)	43.69 (41.7, 45.7)	4/01/2006	22/09/2008	2.7	16.1	16.5
Team Y	17	150.12 (148.9, 151.4)	155.76 (153.7, 157.8)	36.68 (35.2, 38.2)	41.79 (40.5, 43.1)	27/03/2006	5/04/2008	2.0	15.7	10.9
**2008**	**43**	**144.58 (143.8, 145.4)**	**150.07 (149.2, 150.9)**	**34.12 (33.6,34.7)**	**39.35 (38.0,40.7)**	**1/02/2007**	**1/07/2009**	**2.4**	**15.9**	**19.0**
Team X	23	145.44 (144.6, 146.3)	150.28(149.0, 151.5)	34.59 (33.7, 35.5)	40.43 (38.6, 42.3)	9/05/2007	1/07/2009	2.1	12.7	19.0
Team Y	20	143.55 (142.5, 144.6)	149.83 (148.8, 150.8)	33.77 (33.0, 34.5)	38.36 (36.3, 40.4)	1/02/2007	3/02/2009	2.0	14.1	14.6
**2009**	**25**	**138.26 (137.2, 139.3)**	**144.96 (143.9, 146.0)**	**30.62 (29.9, 31.3)**	**35.23 (34.4, 36.0)**	**18/04/2007**	**17/05/2010**	**3.1**	**15.0**	**9.1**
Team X	19	138.80 (137.5, 140.1)	144.95 (143.8, 146.1)	30.93 (30.1, 31.8)	35.55 (34.8, 36.3)	17/05/2008	3/11/2009	1.5	13.9	8.8
Team Y	6	137.33 (134.6, 140.0)	144.13 (140.6, 147.7)	30.03 (28.6, 31.5)	33.70 (31.4, 36.0)	18/04/2007	17/05/2010	3.1	13.5	8.4
**2010**	**23**	**133.13 (131.9, 134.4)**	**139.42 (138.7, 140.2)**	**27.81 (26.5, 29.1)**	**32.44 (31.3, 33.6)**	**10/03/2009**	**5/05/2011**	**2.2**	**12.5**	**12.5**
Team X	4	133.40 (132.9, 134.0)	137.35 (135.8, 138.9)	27.55 (27.5, 27.6)	33.90 (32.0, 35.8)	10/03/2009	25/11/2009	0.7	5.5	7.8
Team Y	19	133.25 (131.6, 134.9)	139.65 (138.9, 140.5)	28.23 (26.7, 29.8)	32.33 (31.0, 33.6)	20/09/2009	5/05/2011	1.6	12.5	12.5
**2011**	**21**	**127.18 (125.9, 128.5)**	**132.05 (130.9, 133.2)**	**24.25 (23.5, 25.0)**	**28.05 (27.3, 28.8)**	**31/10/2009**	**3/04/2012**	**2.4**	**14.5**	**9.1**
Team Y	21	127.18 (125.9, 128.5)	132.05 (130.9, 133.2)	24.25 (23.47, 25.04)	28.05 (27.34, 28.77)	31/10/2009	3/04/2012	2.4	14.5	9.1
**2012**	**13**	**122.14 (120.5, 123.8)**	**125.71 (124.9, 126.6)**	**22.36 (21.8, 22.9)**	**24.86 (23.8, 25.9)**	**8/06/2011**	**12/03/2013**	**1.8**	**8.0**	**6.8**
Team Y	13	122.14 (120.5, 123.8)	125.71 (124.9, 126.6)	22.36 (21.8, 22.9)	24.86 (23.8, 25.9)	8/06/2011	12/03/2013	1.8	8.0	6.8
**2013**	**5**	**115.67 (111.9, 119.4)**	**120.33 (119.8, 120.9)**	**19.47 (18.8, 20.2)**	**21.90 (20.5, 23.3)**	**25/10/2012**	**16/05/2013**	**0.6**	**9.0**	**4.2**
Team Y	5	115.67 (111.9, 119.4)	120.33 (119.8, 120.8)	19.47 (18.8, 20.2)	21.90 (20.5, 23.3)	25/10/2012	16/05/2013	0.6	9.0	4.2
**2014**	**1**	**109.00 (/)**	**109.00 (/)**	18.90 (/)	18.90 (/)	**21/04/2013**	**21/04/2013**	**0.0**	**0.0**	**0.0**
Team Y	1	109.00 (/)	109.00 (/)	18.90 (/)	18.90 (/)	21/04/2013	21/04/2013	0.0	0.0	0.0
**Total**	**302**	**138.83 (137.3, 140.4)**	**166.40 (165.0, 167.8)**	**36.54 (36.3, 36.8)**	**69.95 (68.2, 71.8)**	**16/01/2003**	**16/05/2013**	**1.9**	**12.4**	**18.2**

The age difference was corrected because of reallocation. If a player is older, this player is usually the smallest. And the youngest player is usually the tallest. This is demonstrated in Table 4. The oldest player was born on 18/04/2007 and has a stature of 134 cm (on the growth curves, this stature corresponds with the P50 for almost 9 years old), whereas the youngest player was born on 22/01/2010 but is 147.5-cm tall (on the growth curves, this corresponds with 11-year-olds). Due to this change, a taller player might have a physical advantage over the older player. Obviously, the older player could have enhanced cognitive skills, which in turn could compensate for the stature difference.

**Table 4:** Player distribution of a sampled U11 Belgian academy soccer team, where the tallest player is also the youngest player.

**U11**		
**Birth date**	**Stature**	**Allocation date**
18/04/2007	134.0	15/03/2009
11/01/2008	138.5	21/03/2009
17/05/2008	140.2	7/04/2009
14/08/2008	144.3	21/03/2009
24/11/2008	139.2	11/05/2009
2/01/2009	135.1	29/11/2009
19/01/2009	139.9	26/07/2009
15/02/2009	142.5	6/04/2009
2/03/2009	139.1	28/09/2009
6/03/2009	146.1	31/03/2009
12/03/2009	139.8	3/09/2009
12/03/2009	146.4	2/01/2009
2/04/2009	146.4	15/03/2009
29/04/2009	145.2	13/07/2009
3/05/2009	145.3	28/01/2009
7/06/2009	137.4	16/12/2009
7/06/2009	144.2	2/04/2009
10/07/2009	142.7	2/09/2009
18/07/2009	144.1	22/05/2009
27/07/2009	140.7	11/08/2009
30/07/2009	136.2	12/10/2009
21/10/2009	149.0	22/02/2009
3/11/2009	143.7	15/06/2009
22/01/2010	147.5	19/04/2009

In Table 5, the category changes are displayed that result from this new allocation method. Forty-seven percent of the players would be allocated to a different age category compared with the current one. One percent would be reallocated two age categories lower, 21% would be reallocated one age category lower, 23% would be reallocated one age category higher, and just 2% would be reallocated two age categories higher. For Team X, the percentage of players that are not reallocated is 56%, whereas for Team Y, 51% of the players would not be reallocated. However, the dataset received from Team Y was slightly bigger, so in reality the results can be considered comparable.

**Table 5:** Overview of the category changes that result from the new allocation method, as well as the percentage of players who are not reallocated for ## Belgian academy soccer players.

**Year**	**2 teams down**	**1 team down**	**Same team**	**1 team up**	**2 teams up**	**Total**	**Not reallocated (%)**
**2003**		7	6	4		17	35
Team X		7	5	4		16	31
Team Y			1			1	100
**2004**		9	16	12	1	38	42
Team X		4	9	4	1	18	50
Team Y		5	7	8		20	35
**2005**		7	14	10	3	34	41
Team X		4	9	6		19	47
Team Y		3	5	4	3	15	33
**2006**		8	20	9	1	38	53
Team X		4	7	4	1	16	44
Team Y		4	13	5		22	59
**2007**	1	13	24	10	1	49	49
Team X		4	15	3		22	68
Team Y	1	9	9	7	1	27	33
2008		3	25	9		37	68
Team X		2	15	5		22	68
Team Y		1	10	4		15	67
**2009**	1	9	18	5		33	55
Team X		4	17	4		25	68
Team Y	1	5	1	1		8	13
**2010**		3	10	3		16	63
Team Y		3	10	3		16	63
**2011**		3	15	4		22	68
Team Y		3	15	4		22	68
**2012**		1	9	2		12	75
Team Y		1	9	2		12	75
**2013**		1	4	1		6	67
Team Y		1	4	1		6	67
**Total**	**2**	**64**	**161**	**69**	**6**	**302**	**53**

#### Results (Phase II)

**Figure 4:**
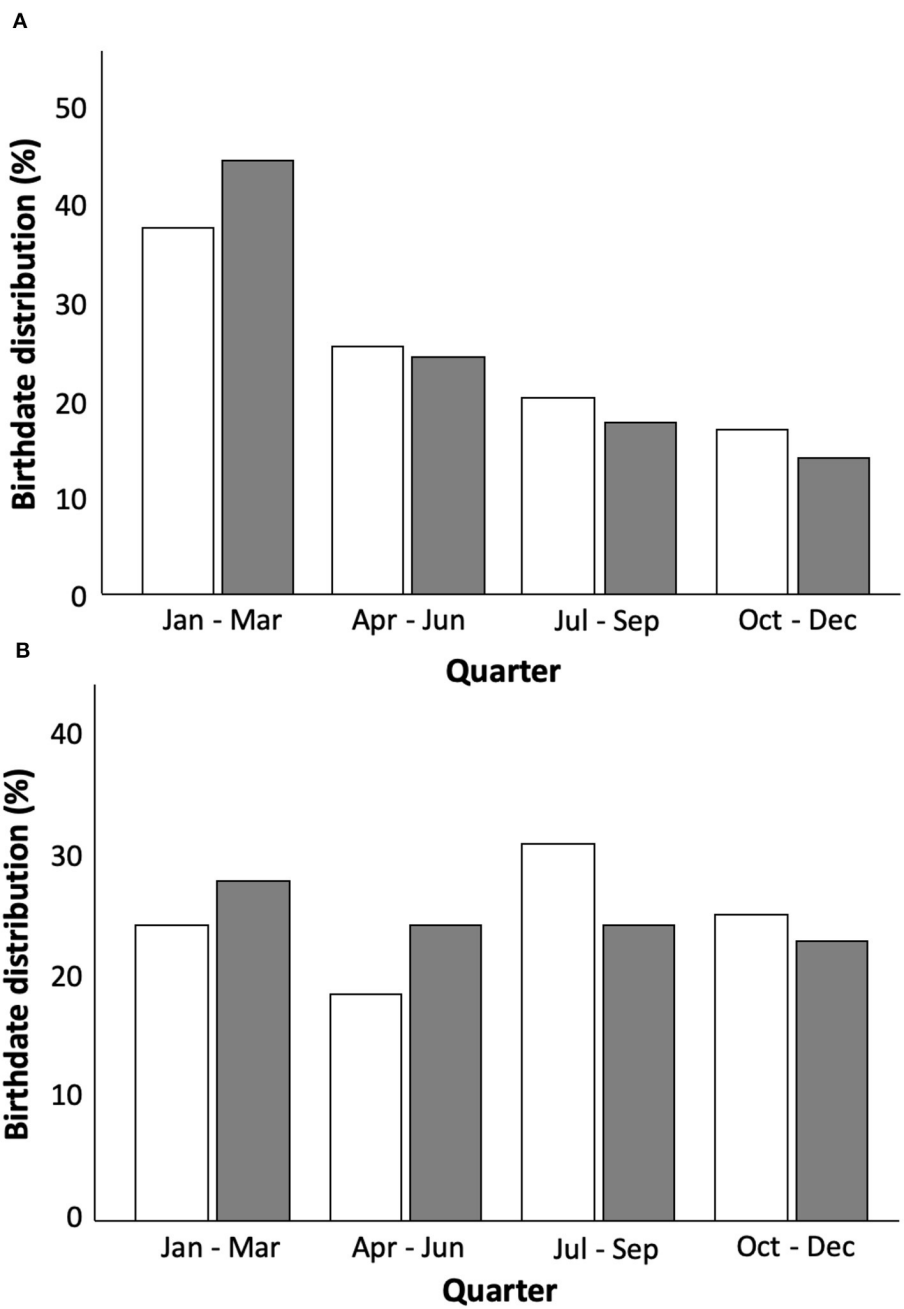
Birthdate distribution of 302 Belgian academy soccer players in percentage per quarter for team X (white bars) and team Y (gray bars), before **(A)** and after reallocation **(B)**.

**Figure 5:**
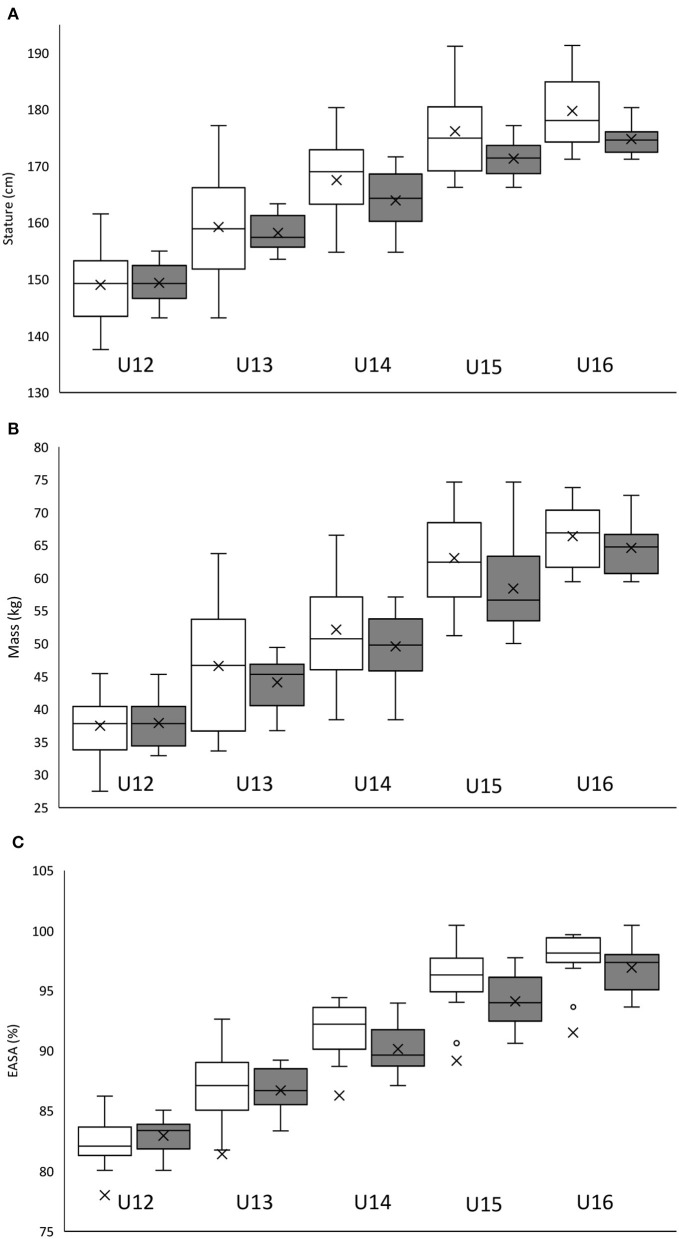
The mean ± SD (95% CI) for anthropometric (**A**: stature; **B**: mass; **C**: estimated percentage of final adult stature attainment) characteristics of 80 UK academy soccer players according to traditional chronologically ordered (white bars) vs. the proposed reallocation method (gray bars).

The mean ± SD (95% CI) and associated effect sizes for physical and anthropometric characteristics according to traditional, chronologically ordered vs. the proposed reallocation method for categorizing players are displayed in Table 6. For the 20 comparisons exploring the impact of the reallocation, group CV was reduced (0.2–10.1%) in 15 metrics across U11 to U16 age categories, with the U13 age category demonstrating the largest reductions (0.9–10.1%) in CV (see Figures 4, 5). The U12 and U13 age categories and associated reallocation groupings showed *trivial* to *small* (ES = 0.0–0.5) between-method differences. With *trivial* to *moderate* (ES = 0.0–1.1) differences within the U14–U16 age categories.

**Table 6:** Summary table of mean ± SD (95% CI) and effect sizes for physical and anthropometrical characteristics of 80 UK academy soccer players (U12–U16) according to traditional chronologically ordered and the proposed reallocation method.

**Banding method**	**Chronological banding**	**Reallocation method**	**Effect size**	**Chronological banding**	**Reallocation method**	**Effect size**	**Chronological banding**	**Reallocation method**	**Effect size**	**Chronological banding**	**Reallocation method**	**Effect size**	**Chronological banding**	**Reallocation method**	**Effect size**
**Age grouping**	**U12**	**U12**	**U12**	**U13**	**U13**	**U13**	**U14**	**U14**	**U14**	**U15**	**U15**	**U15**	**U16**	**U16**	**U16**
	**(n = 7)**	**(n = 7)**		**(n = 11)**	**(n = 8)**		**(n = 15)**	**(n = 11)**		**(n = 12)**	**(n = 11)**		**(n = 11)**	**(n = 10)**	
**Metrics**															
Stature (cm)	149.0 ± 6.2 (146.1–151.8)	149.4 ± 3.6 (147.5–151.2)	0.1, Trivial	159.2 ± 9.9 (146.1–151.8)	158.2 ± 3.3 (155.9–160.5)	0.2, Trivial	167.5 ± 7.3 (163.8–171.2)	163.9 ± 5.3 (160.9–166.9)	0.6, Small	176.1 ± 6.2 (172.7–179.6)	171.3 ± 2.9 (169.8–172.9)	0.9, Moderate	179.8 ± 6.3 (176.6–182.9)	174.7 ± 2.7 (173.1–176.4)	1.1, Moderate
CV (%)	4.2	2.4		6.2	2.1		4.3	3.2		4.1	1.7		3.5	1.5	
Mass (kg)	37.5 ± 6.2 (35.3–39.6)	39.9 ± 3.6 (36.0–39.8)	0.1, Trivial	46.6 ± 9.1 (41.8–51.4)	44.1 ± 4.1 (41.2–46.9)	0.4, Small	52.1 ± 7.8 (48.2–56.1)	49.6 ± 5.6 (46.4–52.7)	0.4, Small	63.0 ± 6.8 (59.8–66.3)	58.4 ± 6.8 (54.8–62.0)	0.7, Moderate	66.4 ± 4.4 (64.2–68.6)	64.6 ± 3.8 (62.2–67.0)	0.4, Small
CV (%)	12.7	9.6		19.5	9.4		15.0	11.3		10.9	11.7		6.7	6.0	
CMJ (cm)	24.9 ± 3.8 (22.1–27.6)	25.8 ± 2.1 (24.3–27.3)	0.3, Small	28.2 ± 3.1 (26.3–30.0)	28.0 ± 2.9 (25.1–30.8)	0.1, Trivial	28.9 ± 4.2 (26.8–30.0)	28.9 ± 4.6 (26.2–31.6)	0.0, Trivial	35.6 ± 4.5 (33.0–38.1)	30.5 ± 4.5 (27.7–33.2)	1.1, Moderate	38.4 ± 4.7 (35.6–41.1)	36.4 ± 3.4 (34.2–38.6)	0.5, Small
CV (%)	15.1	8.1		11.0	10.3		14.5	15.7		12.7	14.9		12.3	9.2	
COD (sec)	2.8 ± 0.1 (2.7–2.9)	2.7 ± 0.1 (2.7–2.8)	0.4 Small	2.7 ± 0.1 (2.6–2.7)	2.7 ± 0.1 (2.6–2.8)	0.1, Trivial	2.6 ± 0.1 (2.5–2.6)	2.6 ± 0.1 (2.6–2.7)	0.5, Small	2.5 ± 0.1 (2.5–2.6)	2.5 ± 0.1 (2.5–2.6)	0.2, Trivial	2.4 ± 0.1 (2.4–2.5)	2.4 ± 0.1 (2.4–2.5)	0.3 Small
CV (%)	4.4	3.6		4.0	3.4		5.4	5.0		3.7	4.8		3.4	2.1	
5m (sec)	1.1 ± 0.1 (1.1–1.1)	1.1 ± 0.0 (1.1–1.1)	0.0, Trivial	1.1 ± 0.0 (1.0–1.1)	1.1 ± 0.0 (1.1–1.1)	0.4, Small	1.0 ± 0.1 (1.0–1.1)	1.1 ± 0.0 (1.0–1.1)	0.0, Trivial	1.0 ± 0.0 (0.9–1.0)	1.0 ± 0.1 (1.0–1.0)	0.9, Moderate	1.0 ± 0.0 (1.0–1.0)	0.9 ± 0.0 (0.9–1.0)	1.0, Moderate
CV (%)	5.5	4.2		3.4	3.3		5.3	3.9		4.6	5.0		4.8	3.1	
10m (sec)	1.9 ± 0.1 (1.9–2.0)	1.9 ± 0.1 (1.9–2.0)	0.4, Small	1.9 ± 0.1 (1.8–1.9)	1.8 ± 0.0 (1.8–1.9)	0.0, Trivial	1.8 ± 0.1 (1.8–1.9)	1.9 ± 0.1 (1.8–1.9)	0.8, Moderate	1.7 ± 0.1 (1.7–1.8)	1.8 ± 0.1 (1.7–1.8)	0.3, Small	1.7 ± 0.1 (1.7–1.8)	1.7 ± 0.1 (1.7–1.7)	0.1, Trivial
CV (%)	4.0	3.1		3.4	2.0		5.0	4.2		3.6	4.2		5.0	3.1	
20m (sec)	3.5 ± 0.2 (3.3–3.6)	3.4 ± 0.1 (3.3–3.5)	0.4, Small	3.3 ± 0.1 (3.2–3.3)	3.3 ± 0.1 (3.2–3.4)	0.0, Trivial	3.2 ± 0.1 (3.2–3.3)	3.3 ± 0.1 (3.2–3.3)	0.6, Small	3.0 ± 0.1 (3.0–3.1)	3.1 ± 0.1 (3.1–3.2)	0.7, Moderate	2.9 ± 0.1 (2.9–3.0)	2.9 ± 0.1 (2.9–3.0)	0.2, Small
CV (%)	5.8	3.6		2.9	2.3		3.8	3.4		4.2	3.7		4.3	3.7	
EASA (%)	82.6 ± 1.6 (81.8–83.3)	82.9 ± 1.4 (82.2–83.7)	0.2, Small	87.2 ± 2.7 (85.8–88.6)	86.7 ± 1.9 (85.4–88.0)	0.2, Small	92.0 ± 1.8 (91.1–93.0)	90.2 ± 2.0 (89.0–91.3)	0.9, Moderate	96.6 ± 2.6 (95.2–98.1)	94.1 ± 2.2 (92.9–95.3)	1.1, Moderate	98.1 ± 1.6 (97.2–98.9)	96.9 ± 2.1 (95.5–98.3)	0.6, Small
CV (%)	1.9	1.7		3.1	2.2		2.0	2.2		2.7	2.3		1.6	2.2	

*Under (U); Coefficient of variance (CV); Effect size (ES); Estimated adult stature attainment (EASA); Change of direction (5-0-5 test)*.

*Effect size thresholds: trivial = <0.20; small = >0.21–0.60; moderate = >0.61–1.20; large = >1.21–2.00; very large = >2.01*.

## Discussion

The primary aim of this study was to examine to what extent a new method of player allocation based on an ED age, using the midway point of the chronological and developmental birth dates, could provide a solution to decrease and, ultimately, to solve the RAE and maturity-related bias. With the use of two separate academy soccer player datasets, the new allocation method was explored. The findings of the present study were four-fold: (1) the current age distribution per team and per quartile was similar to the distribution in literature and clearly showed a RAE for the entire sample of players (Barnsley et al., 1985; Verhulst, 1992; Helsen et al., 1998, 2000, 2012; Hurley et al., 2001; Musch and Grondin, 2001; Cobley et al., 2009; Nolan and Howell, 2010; Christina Steingröver et al., 2017a); (2) the RAE was also calculated for aggregate age groupings (e.g., U13 and U14), and in almost all groups (except U11–U12), a RAE was present; (3) with the use of the new allocation method, 47% (*n* = 141) of players would have been allocated to a different age playing category compared with the current system. (4) Fifteen of the 20 (75%) observed between-method comparisons show that the reallocation method reduced group CV, which was consistent for anthropometric and maturation status characteristics for all of the sampled age categories. Given the two-part design of the study, we will now discuss these findings separately beginning with phase I.

### Phase I: Examination of the Midway Point Between the Chronological and Estimated Developmental Birth Dates to Reallocate Youth Players

Regarding the first point, the magnitude of the RAE was a factor of “four” for the overall sample of youth players (Q1 = 41.4 vs. Q4 = 14.9%). Specifically, this means that there were four times more players born in the first quarter compared with the last quarter. Second, examining the aggregated age groupings (i.e., U7–U8 and U9–U10), again, a RAE was present compared with the population norm distributions (Q1 = 44.4 and 36.8%; Q4 = 5.6 and 10.5%) except for the U11–U12 group (Q1 = 35.7%; Q4 = 18.6%). The latter finding can be explained by the smaller sample size of the youngest age groups that was <50% of the other groups. With respect to the newly proposed reallocation method, the midway point was used between the chronological and ED birth dates that were calculated using the normative growth curves of a study examining secular changes in biological maturation in Belgian boys of the same age categories (Roelants et al., 2009). Before reallocation, the mean differences in player stature and body mass were 24.0 cm and 20.1 kg, respectively. After reallocation, these differences were reduced to 12.4 cm and 18.2 kg, respectively, possibly due to players being more closely matched to stage of biological maturity. With the use of this new method, for almost half (53%; *n* = 161) of the players, a reallocation to another team was not recommended. Specifically, 47% (*n* = 141) of the players in the current selection system are reallocated to a different age category. Of this group of movers, 1% would be reallocated two age categories lower, 21% one age category lower, 23% one age category higher, and 2% two age categories higher. As a result, their skills to compete with their counterparts will be enhanced due to the fact that the variance in stature/body mass has been decreased within the reallocation group. This demonstrates that reallocation method is easy to implement from a practical point of view, given that the total number of players in each category remains almost the same. Obviously, we need to consider other issues that are linked to moving players to a category higher or lower, for instance, social (e.g., friends playing in a different team) or psychological (i.e., youth players with a different cognitive compared with physical maturation). This might be the case if athletes are dropped to “younger” age groups. Proper communication between the coaching staff, the player, and his/her parents with respect to the importance of this reallocation for appropriate long-term player development is certainly recommended.

### Phase II: Assessment of the Within-Group Variation of Somatic and Physical Fitness Characteristics Using Chronological and Estimated Developmental Birth Dates

As well as being cost-efficient and easy to apply, the present study (phase II) also provides promising evidence to suggest that the newly proposed player (re)allocation method is seemingly an appropriate strategy for reducing transient, maturity-related anthropometric (physical fitness to a lesser extent) characteristics, which are often afforded to early maturing players, who can also be relatively older (Carling et al., 2009; Towlson et al., 2017). This is evidenced by 75% of the observed between-method comparisons showing that the reallocation method reduced group CV, which was consistent for anthropometric and maturation status characteristics for all of the sampled age categories. This is of relevance and importance for soccer practitioners given that maturity and relative age selection bias can contribute to the premature deselection and playing position allocation of academy soccer players (Towlson et al., 2017), which ultimately confounds the (de)selection processes of talent development centers across the world and likely limits the size of the talent pool for clubs and nations to select from. Therefore, this study provides persuasive early evidence for the application of a new player allocation method to remove the temporary, physical fitness, and anthropometric advantages afforded to older (and sometimes more mature) players.

### Limitations of the Newly Proposed Reallocation Model

Despite demonstrating early promise, the newly proposed reallocation method is not without limitations. For instance, the ED birth date for the reallocation method was calculated using the growth curves, which are based on a study published in 2004 or 16 years ago (Roelants et al., 2009). The idea of generic, worldwide normative growth curves has been addressed upon request of the World Health Organization (Beunen et al., 2006), but not realized yet. Therefore, the curves used in this study as a reference point are perhaps limited and only contain growth data of Belgian children and adolescents. More recent (longitudinal) data may have a slightly different impact on the estimations. The second discussion point is that this study considered a specific target group of academy soccer players. In contrast, the growth curves were based on a broader population of mixed ethnicity. This issue could be solved by creating specific growth curves for youth players, eventually even per sport.

Also, despite the fact that academy soccer practitioners suggest that players' physical, maturity, and relative age characteristics are not considered during players' talent selection (Towlson et al., 2019), temporary maturity-related enhancements in anthropometric and physical fitness characteristics seemingly remain to be a consistent discriminatory factor for players who are (de)selected for talent programs (Lovell et al., 2015) and indeed allocated certain playing positions (Deprez et al., 2015; Towlson et al., 2017). Given this constant (sub)conscious maturity-related selection bias, it is important to acknowledge that although the present study has shown that the between-group variation in maturation status does reduce within the reallocation groupings, it is unknown whether reallocated players will mature at a rate that is consistent with that of their new peers.

### Future Directions for Research

Although the present study sampled players from two professional soccer academies, which created a combined dataset of 302 academy soccer players for the initial player reallocation (phase I), we acknowledge that further investigations using our newly proposed player allocation method are required to better understand the efficacy of our method to reduce temporary, maturity-related differences between players. Therefore, practitioners and governing bodies should take a coordinated approach to player development research and work collaboratively to aggregate players' relative age, maturity, physical fitness, and anthropometric datasets in order to truly understand the impact of both relative age and maturity selection bias and to offer insight on the effectiveness of new approaches to remove such selection bias. Finally, in this newly proposed reallocation method, we used the midway point between the chronological and ED birth dates. It may be considered to give more weight to either the chronological or developmental birth date, although we now contend the midway point as more intuitive for coaches and practitioners. As well, we only considered physical maturation, while cognitive maturation may also play a role in the reallocation process of youth players, as psychological factors are considered a priority by practitioners during the talent selection process (Towlson et al., 2019). Finally, this study provides a one-time snapshot of RAE across ages at one point in time. It cannot address the insidious effects that RAE has inevitably had on athlete success and or dropout leading up to this moment in time, or how RAE may have differentially affected different age groups prior.

**Figure 6:**
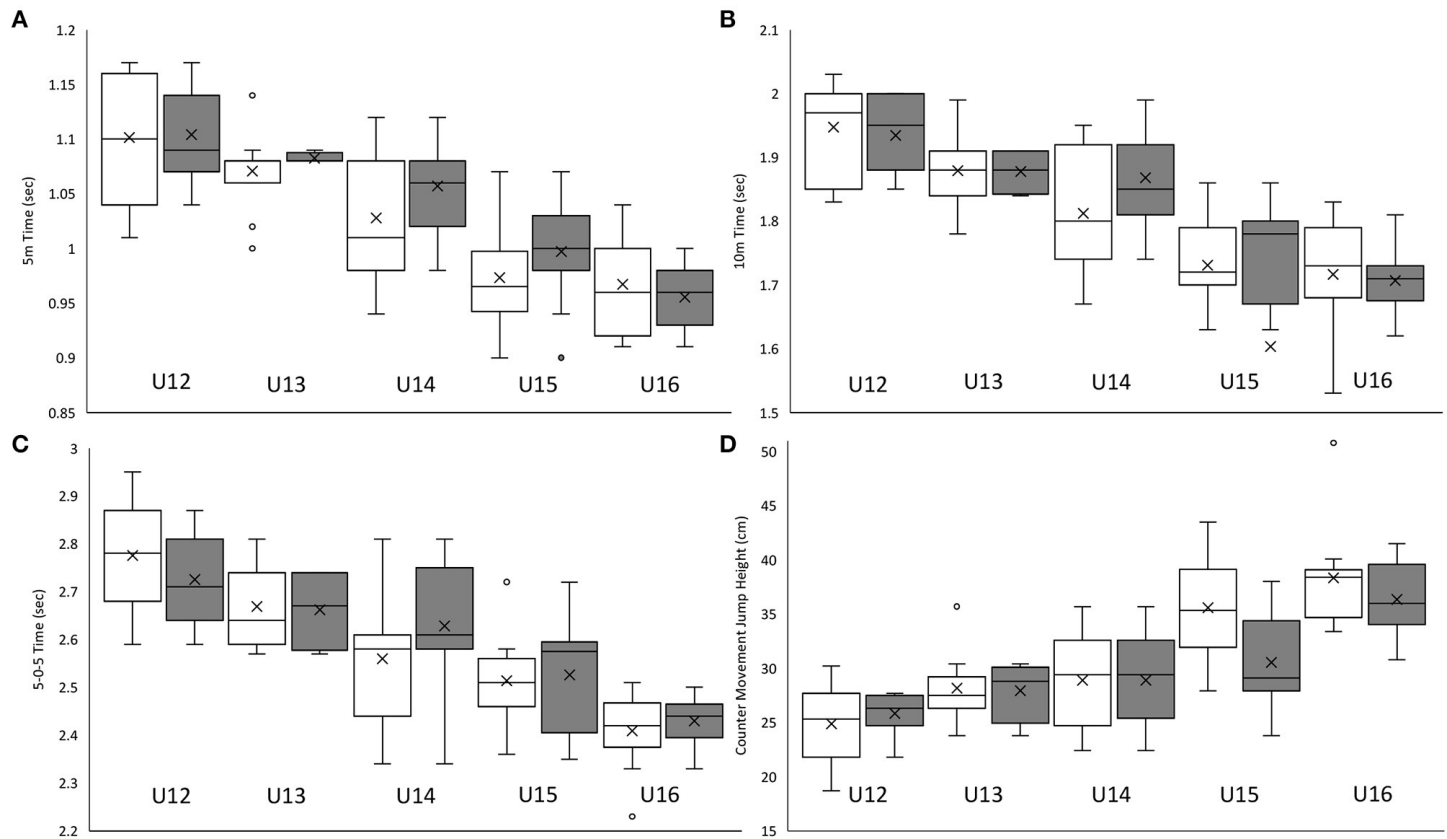
The mean ± SD (95% CI) for physical (**A**: 5 m acceleration time; **B**: 10 m acceleration time; **C**: 5-0-5 time; **D**: counter movement jump height) characteristics of 80 UK academy soccer players according to traditional chronologically ordered (white bars) vs. the proposed reallocation method (gray bars).

Although in its infancy, this new player allocation method shows early promise to reduce the over-representation of players born within a particular quartile (i.e., quartiles 1 and 2). As well, the findings of the present study suggest that birth date distribution would become more evenly spread and age differences reduced within the newly formed allocation grouping. Generally speaking, the younger player of one category will likely be the smallest (or one of the smallest), and the oldest player is likely the tallest (or one of the tallest). In fact, this is a particular benefit of using the median point between chronological and ED birth dates, given that if a player does not show a stature corresponding to his age, his ED birth date will compensate for this (Figure 6). Therefore, being the oldest player may not provide a physical advantage to this player, as is the case in the current selection procedure. In return, however, older players gain a cognitive advantage, which is not considered in the actual selection system today.

### Immediate Practical Implications

Regarding implementation, the best period to measure may be the end of the season in order to give clubs proper time to compose the youth categories for the next season. Along the same lines, we consider one measurement per season during growth spurt appropriate in order to avoid a reorganization in-season. Finally, we do not consider this reallocation as a disadvantage for technical or tactical periodization if players are moved up or down. The first priority is the long-term player development, and tactical skills can also be taught at a later stage.

There may be particular implications for athletes being dropped to “younger” age groups and potentially being “re-shuffled” the subsequent years. This may impact their social relationships, as they are no longer competing in the same team as their friends or schoolmates. Despite the prospect of later-developing athletes being recategorized to participate in “younger” chronologically ordered age groups, “bio-banded” athletes have reported that they enjoyed and understood the purpose of the format, while feeling that there was less chance of sustaining injury (Cumming et al., 2018). Also, recategorized athletes have also reported that development-based categorization methods provided more opportunity for them to engage key psychological constructs (Cumming et al., 2018), deemed important when assessing talent (Towlson et al., 2019). In addition, parents of recategorized athletes have stated that they trusted coaches to do what is right for their child's development and that such methods would not be adopted if the staff did not believe there was any value in doing so (Reeves et al., 2018). Apart from physical development, there may also be other reasons to reallocate players to a younger age group such as cognitive maturity that cannot be captured with this new method. In some countries, there is indeed a similar “medical dispensation” procedure to reallocate players to a younger age group because of neurological issues such as attention deficit hyperactivity disorder (ADHD) or autism spectrum disorders. In any case, proper communication between the coaching staff, the player, and his/her parents with respect to the importance of this reallocation for appropriate long-term player development is certainly recommended.

There are also practical considerations (and directions) for sports governing bodies looking to adopt this method. Rather than leaving the initiative to individual clubs, it is much more appropriate to look for a structural solution that is implemented by the national governing bodies for a given level of competition where teams are involved in (e.g., all age categories in elite youth soccer).

## Conclusion

In summary, in this study, an innovative and evidence-based allocation method that is easy to implement was proposed and tested (given few personnel and little funding is required). With the use of a dataset of 302 academy soccer players, the results first of all showed that this new allocation method can level the playing field with respect to stature and body mass differences and also result in a more even distribution of birth dates throughout a selection year. In fact, while a clear RAE was found for the overall sample of youth players before reallocation, it completely disappeared after reallocation. Second, the examination of 80 UK academy soccer players also confirmed that reallocating players based upon development age reduced significantly the within-playing group variation of both somatic and physical fitness characteristics. The reduction in relative age- and maturity-related bias will lead to fewer dropouts and thus a larger player pool, which could benefit, in turn, talent detection, selection, and development. Put differently, this new method allows the retention of as many youth players as possible, for as long as possible, in the best learning environment possible.

## Data Availability Statement

The raw data supporting the conclusions of this article will be made available by the authors, without undue reservation.

## Ethics Statement

The studies involving human participants were reviewed and approved by Informed assent and parental consent were acquired for each player prior to testing and a detailed protocol (MP013675) was approved by the Research Ethics Committee UZ/KU Leuven, Belgium. Written informed consent to participate in this study was provided by the participants' legal guardian/next of kin.

## Author Contributions

WH initiated the research from the very beginning, transferred this idea to an online application that can be used by national associations, and clubs considering to reallocate their youth players to a higher or a lower team that is more in line with their physical development. CT was also involved from the start and contributed to all stages of the study. He was assisted by CM, in particular for the statistics and the processing of the UK data. JS and MT revised preliminary drafts of the paper as well as the final version. SV contributed to the study as it was part of hismaster's thesis. GO is a practitioner who is involved in the relative age and late maturity effects for many years, launched the idea for calculation of the developmental age as highlighted in the study (inverse use of growth curves), and contributed to the customized excel sheet that was used to produce an automatic developmental age calculator.

## Conflict of Interest

The authors declare that the research was conducted in the absence of any commercial or financial relationships that could be construed as a potential conflict of interest.
